# Feasibility and acceptability of virtually coaching residents on communication skills: a pilot study

**DOI:** 10.1186/s12909-021-02936-w

**Published:** 2021-09-29

**Authors:** Marzena Sasnal, Rebecca Miller-Kuhlmann, Sylvia Bereknyei Merrell, Shannon Beres, Lucas Kipp, Sarah Lee, Zachary Threlkeld, Aussama K. Nassar, Carl A. Gold

**Affiliations:** 1 Stanford-Surgery Policy Improvement Research and Education Center (S-SPIRE), Department of Surgery, Stanford University School of Medicine, 1070 Arastradero Rd, Stanford, CA 94305 USA; 2 Department of Neurology & Neurological Sciences, Center for Academic Medicine, 453 Quarry Road, Stanford, CA 94305 USA; 3 Department of Surgery, Stanford University School of Medicine, 300 Pasteur Dr, Stanford, CA 94305 USA

**Keywords:** Telehealth, Virtual coaching, Communication skills, Resident education, Resident curriculum

## Abstract

**Background:**

Developing communication skills is a key competency for residents. Coaching, broadly accepted as a training modality in medical education, has been proven a successful tool for teaching communication skills. Little research is available thus far to investigate virtual coaching on communication skills for telemedicine encounters. The purpose of the study was to test the hypothesis that virtually coaching residents on communication skills is feasible and acceptable. We surveyed 21 resident-faculty pairs participating in a “fully virtual” coaching session (patient, coach, and resident were virtual).

**Methods:**

We asked 50 neurology resident-faculty coach pairs to complete one “fully virtual” coaching session between May 20 and August 31, 2020. After each session, the resident and coach completed a 15-item survey, including Likert-style scale and open-ended questions, assessing feasibility and acceptability. Descriptive statistics and qualitative content and thematic analyses were performed.

**Results:**

Forty-two percent (21/50) of all eligible residents completed “fully virtual” coaching sessions. The overall survey response rate was 91 % (38/42). The majority of respondents agreed that the direct observation and debriefing conversation were easy to schedule and occurred without technical difficulties and that debriefing elements (self-reflection, feedback, takeaways) were useful for residents. Ninety-five percent of respondents rated the coach’s virtual presence to be not at all disruptive to the resident-patient interaction. Virtual coaching alleviated resident stress associated with observation and was perceived as an opportunity for immediate feedback and a unique approach for resident education that will persist into the future.

**Conclusions:**

In this pilot study, residents and faculty coaches found virtual coaching on communication skills feasible and acceptable for telemedicine encounters. Many elements of our intervention may be adoptable by other residency programs. For example, residents may share their communication goals with clinic faculty supervisors and then invite them to directly observe virtual encounters what could facilitate targeted feedback related to the resident’s goals. Moreover, virtual coaching on communication skills in both the in-person and telemedicine settings may particularly benefit residents in challenging encounters such as those with cognitively impaired patients or with surrogate decision-makers.

**Supplementary Information:**

The online version contains supplementary material available at 10.1186/s12909-021-02936-w.

## Background

Residents frequently participate in difficult encounters, delivering serious news or discussing treatment options [1], and they express a pressing need for communication training [1–4]. Communication skills, recognized by The Accreditation Council for Graduate Medical Education (ACGME) as a core competency [5] can be successfully developed through coaching [6, 7], traditionally occurring with both the coach and coachee physically present in the same location [8]. However, COVID-related restrictions fortified the need for formal integration of virtual coaching into residency training – a format with utility anticipated to outlast the pandemic [9].

Although many health care systems utilized telemedicine before the COVID-19 pandemic [10], its use exploded after March 2020. In response to the pandemic, many medical institutions rapidly transitioned from in-person healthcare delivery and medical education to telehealth, including developing rapid telemedicine curricula [11] or creating new virtual opportunities for trainees to continue their professional growth despite adverse circumstances [12, 13]. Online teaching has enabled the continuation of medical education [14]. For instance, according to the survey conducted in Physical Medicine and Rehabilitation, 92.5 % of residents participated in virtual didactics due to pandemic [15].

Revisiting traditional routines in medical education and adaptation to new telemedicine practices expanded educational opportunities for trainees, increased access to tools and innovations, and enhanced collaboration between providers from different locations [15]. Telemedicine may increase access to care, limit the digital divide, and simplify longitudinal management of chronic conditions [16]. Telehealth is also a feasible and effective tool to screen patients before they reach a hospital and increase clinical volume during the COVID-19 pandemic [17, 18]. Studies conducted at our institution found that telemedicine encounters are largely considered acceptable by clinicians and are more convenient for patients than in-person visits [19, 20]. For these reasons, telemedicine is anticipated to remain a permanent supplement to in-person visits [19, 20]. Training residents to effectively care for patients through telemedicine is a critical need.

Virtual coaching in medicine has been utilized primarily for coaching patients on interventions such as diabetes management [21], weight management [22–24], and cardiac rehabilitation [25, 26]. In medical education, virtual coaching has been used to promote development of technical skills, such as surgical techniques [27–30]. To our knowledge, no existing studies have investigated virtual coaching on communication skills nor coaching in the setting of telemedicine encounters, which by their nature depend on effective verbal communication.

We conducted a pilot study to evaluate the feasibility and acceptability of virtually coaching neurology residents on communication skills for telemedicine patient encounters and to explore how those encounters occur [31]. We also described elements of the intervention that other residency programs may adapt.

## Methods

### Study design and setting

The Stanford Neurology Residency Communication Coaching Program was developed in response to a needs assessment to support communication goals identified by our residents [4]. This program was modeled on the Stanford Pediatrics Residency Coaching Program with an emphasis on communication skills [32]. Four faculty members received protected time to serve as communication coaches. The coaches participated in an intensive half-day orientation followed by monthly 90-minute faculty development sessions focusing on resident communication and coaching skills. The coaches and residents completed a foundational course in relationship-centered communication meant to create a shared communication framework. Course topics include establishing rapport, exploring patients’ understanding of their conditions, and responding to emotions (see Additional file 4 for faculty development curriculum). Resident input was incorporated throughout the development of the coaching program and the faculty coach selection process.

Each of the 50 neurology residents (36–39 in a given academic year; 50 total in this study due to academic year turnover) was paired with a coach and was expected to have 5–8 coaching sessions annually. In the pre-pandemic design, a coaching session was planned to involve in-person observation of the resident-patient interaction in an ambulatory or inpatient setting, followed by in-person debriefing during which the coach would facilitate the resident’s self-reflection on the communication performance, provide targeted feedback, and guide the resident to identify a communication-related takeaway to be practiced before the next coaching session. The faculty coaches were not part of the health care team and not responsible for evaluating resident performance.

The coaching program was set to launch in March 2020. This coincided with the pandemic’s onset, making in-person observations infeasible. At a series of morning report sessions and via email, residents shared their enthusiasm for continuing the coaching program despite the challenges of the pandemic. As resident ambulatory clinic encounters shifted rapidly to telemedicine, we pivoted the coaching program virtually to support resident communication skills within this new healthcare delivery method.

**Fig. 1:**
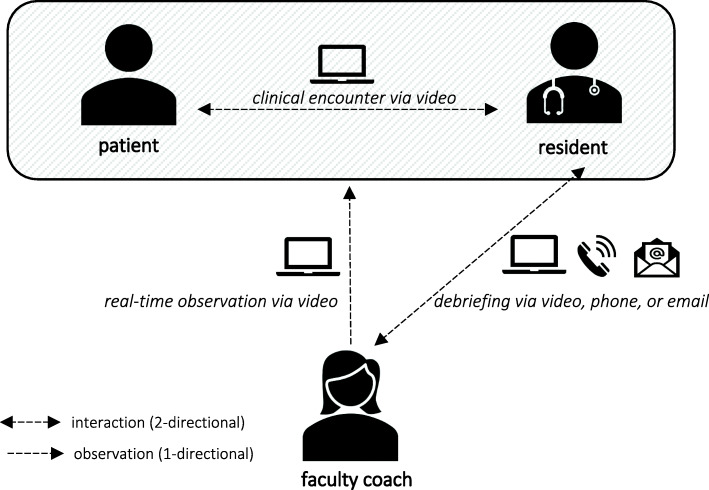
Conceptual Model of “Fully Virtual” Coaching Session. Legend: A “fully virtual” coaching session involved: a real-time observation of the resident-patient telehealth clinical encounter by the coach, followed by resident-coach debriefing via video, phone, or email, during which the coach facilitated the resident’s self-reflection on the communication performance, provided targeted feedback, and guided the resident to identify a communication-related takeaway to be practiced before the next coaching session. Residents conducted clinic visits using secure Zoom software. The coach joined the same Zoom session as the resident and patient, introduced themselves to the patient, and then turned off video and audio and observed the resident-patient interaction

We asked all 50 resident-coach pairs to pilot one “fully virtual” coaching session between May 20 and August 31, 2020. Fully virtual coaching sessions met the following criteria: resident-patient clinical encounter by video; real-time observation by coach of resident-patient communication via video; debriefing conversation between coach and resident by video, phone, or email (Fig. 1). Residents conducted clinic visits using secure Zoom software. The coach joined the same Zoom session as the resident and patient, introduced themselves to the patient, and then muted audio and turned off video to unobtrusively observe the resident-patient interaction. After each coaching session, a 15-item survey was distributed via a secure personalized link to both the coach and the resident (see Additional files 1 and 2). Data were de-identified prior to analysis.

### Measures

Survey included 5-point Likert-style scale questions to assess the feasibility of scheduling and executing direct observations and debriefing conversations, the acceptability of the coach’s presence as an observer, and the perceived usefulness of the three elements of the debriefing conversation: facilitated self-reflection, feedback, and takeaways. Three open-ended response questions explored attitudes, challenges, and benefits related to virtual coaching on telemedicine encounters.

### Data analysis

We performed descriptive statistical analysis of Likert-style scale questions (Excel, Microsoft Corp., Redmond, Washington, USA). Distributions were stratified according to survey respondent type (coach/resident). Free-text responses were coded and analyzed in NVivo (Release 1.3, QSR International Pty Ltd.), resulting in code frequencies for content analysis and emergent themes for subsequent in depth thematic analysis [33].

Stanford University determined this study to be exempt from institutional review.

## Results

Our pilot study included 21 fully virtual coaching sessions (completed by 42 % of resident-coach pairs). Barriers for residents who did not complete a virtual coaching session during the study were primarily logistical (e.g., senior residents with few remaining clinical encounters in the 6 weeks between study launch and graduation; residents assigned to clinics focused on in-person encounters; rotations occurring at night or off site, research rotations, or vacation). A lower-than-expected participation rate may be related to the departmental efforts concentrated on urgent patient care in the face of the global pandemic and coaching being a temporarily lower priority at this time. The overall response rate was 91 % (38/42), with 19/21 residents responding and the four coaches completing the survey for 19/21 encounters. All 21 coaching sessions were in the ambulatory neurology setting, with the majority in the resident continuity clinic.

**Fig. 2:**
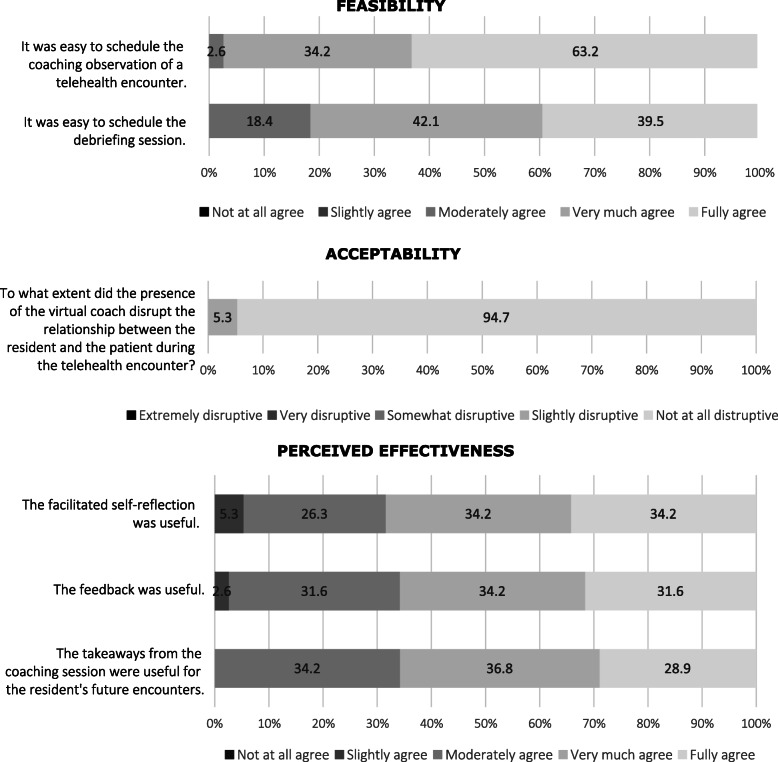
Feasibility, Acceptability and Perceived Effectiveness of Telecoaching on Virtual Encounters – Residents and Coaches’ Perspectives [*n* = 38]

Virtual coaching sessions were reported as feasible by residents and coaches participating in the intervention. The majority of respondents “fully” or “very much” agreed that the direct observation was easy to schedule and they did not experience any technical difficulties during the direct observation (respectively 82 %, *n* = 31 and 87 %, *n* = 33). Similarly, nearly all (97 %, *n* = 37) “fully” or “very much” agreed that the debriefing session was easy to schedule (Fig. 2; findings differentiated between residents and coaches are presented in Additional file 3). Debriefing sessions occurred by video (*n* = 23), phone (*n* = 8), or email (*n* = 4). Seven respondents (28 %) reported some technical issues: two (5 %) reported switching from video to phone due to technical difficulties and five (13 %) reported unreliable internet connection or equipment malfunction.

Nearly all respondents (95 %, *n* = 36) rated the coach’s presence as an observer “not at all” disruptive. Moreover, all or nearly all respondents “fully”, “very much,” or “moderately” agreed that debriefing elements were useful, including facilitated self-reflection (100 %, *n* = 38), feedback (98 %, *n* = 37) and takeaways for future telemedicine encounters (94 %, *n* = 36 moderately).

Qualitative content analysis initially identified five prominent categories related to feasibility and acceptability: logistics; technology; coach’s presence; feedback; and characteristics of virtual coaching. Deeper thematic analysis resulted in themes described in Table 1.

**Table 1:** Residents’ and coaches’ perceptions of virtual coaching on telemedicine encounters: qualitative themes and categorical frequencies

Qualitative Themes	Categorical Labels no. (%) (*n* = 82^a^)	Exemplary Quotes	Comments
***Virtual Coaching Minimizes Logistic Issues with More Flexibility***	***Logistics*** 10 (12.2 %)	- “Easy scheduling, opportunity for immediate feedback. Easily accessible as people can be in different locations. Saves time on transportation.” [Resident] - “The virtual nature of the visits reduces overall time burden for coaches.” [Coach]	Telemedicine encounters and virtual coaching sessions were considered easier to arrange and offered more flexibility for participants in different locations than in-person coaching, with reduced time spent on transportation.
***Technical Skills are Necessary in Virtual Coaching***	***Technology*** 16 (19.5 %)	- “[The challenges are] mostly technical challenges of having multiple people on a Zoom call when the patient is not savvy.” [Resident]	Technical problems and poor technical capabilities of participants were perceived as the biggest challenges of virtual coaching.
***Coach’s Presence as “Fly on the Wall” Observer Possible with Virtual Coaching***	***Coach Presence*** 23 (28.0 %)	- “It is easier to ignore the coach’s presence on televisit than in person, which is helpful. [Resident] - “Far less intrusive than being physically present, potentially minimizing the distraction to the patient and trainee of having a third person in a room and allowing a more “natural” conversation to unfold.” [Coach]	The coach could “truly disappear into the background during observations” [Coach]. The coach was repeatedly referred to as a “fly on the wall.” Virtual observations were described as “more natural” and “less stressful” than in-person observations.
***Virtual Coaching Promotes Feedback***	***Feedback*** 12 (14.6 %)	- “I really appreciated the observation and feedback. It really helped me to better understand my communication strengths and weaknesses. It was really helpful to debrief shortly thereafter.” [Resident] - “Just one session was immensely valuable in helping me make several small changes to my telehealth visits that I am still doing.” [Resident]	Virtual coaching was seen as an opportunity for immediate feedback; feedback was perceived as useful, particularly in the area of verbal communication.
***Innate Qualities of Telemedicine Enhanced by Virtual Coaching***	***Characteristics of Virtual Coaching*** 21 (25.6 %)	- “Challenges encountered in a video visit does not always translate to in-person visits and vice versa. I think it would still be helpful to have in-person coaching in addition to the video session.” [Resident] - “Telehealth encounters have unique communication barriers […] that also need to be taught / optimized.” [Coach]	Virtual coaching was perceived as a unique approach, distinctive from in-person coaching. Also, participants foresaw telemedicine to be commonly used in the future, therefore worth learning skills to make successful.

^a^Number represents the number of categorical sentiments expressed by respondents to the open-ended questions that were included in the qualitative analysis

The virtual coaching sessions were perceived as time-effective, easy to arrange, and offered more flexibility than in-person coaching. Occasional technical difficulties resulted from connection issues or poor technical skills of participants were the primary barrier to virtual coaching. The coach’s virtual presence was seen as less intrusive and disruptive than an in-person observation. A coach observing with video and audio off was barely noticed, which felt “more natural” and alleviated resident stress associated with observation. Participants appreciated immediate and useful feedback. Although virtual coaching on telemedicine encounters was a great opportunity to enhance verbal skills, participants found it challenging for coaches to observe body language in the way that they might during an in-person encounter. Additionally, virtual debriefing sessions did not facilitate the same degree of relationship-building between coaches and residents.

**Fig. 3:**
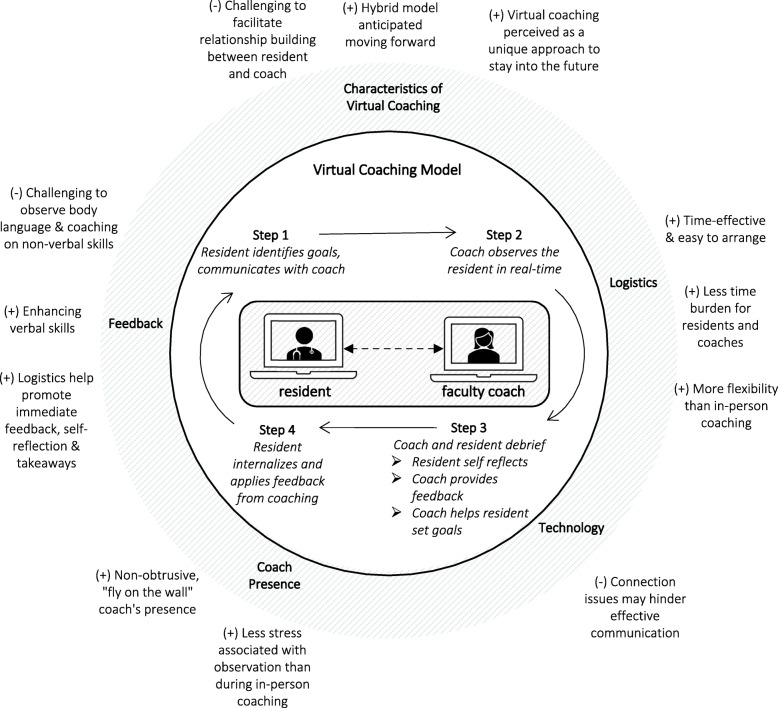
Mechanisms of a Virtual Coaching Model - Benefits and Challenges in Practice. Legend: (+) indicates benefits of virtual coaching sessions; (-) indicates challenges of virtual coaching sessions

With its distinctive challenges and opportunities, coaching in the virtual setting was perceived as a unique approach for resident education that will persist into the future. Participants anticipated a hybrid model moving forward. Participants, even those who were initially skeptical about virtual coaching, found the program both feasible and acceptable: “*I was initially skeptical of whether this would be a useful format to receive coaching, given there are limitations to interpersonal interaction with the patient through video. But the session turned out very helpful, it felt natural, was not disruptive at all, and I received good feedback from my coach*” [Resident]. Benefits and challenges of virtual coaching model identified during thematic analysis are also summarized in Fig. 3.

## Discussion

In this pilot study, neurology residents and their faculty coaches found virtual coaching sessions on telemedicine encounters feasible, acceptable, useful, and easy to schedule. Participants found the coach’s virtual presence as an observer not to be disruptive to the clinical encounter, even suggesting that the coach being “a fly on the wall” during telemedicine encounters is “less intrusive” than in-person clinical encounters. Although the logistics and less intrusive direct observation support sustaining virtual coaching sessions, residents and coaches also desire in-person coaching sessions to enhance the patient-coach relationship and encourage feedback on non-verbal communication.

Coaching of residents by specially-trained faculty has been associated with positive outcomes in other specialties. In pediatrics, residents felt faculty coaches provided higher-quality feedback and incorporated more self-reflection and goal setting than non-coaches and coaches demonstrated increased confidence in delivering feedback on communication skills and goal setting compared to non-coaches [32]. Positive psychology coaching has been associated with reduced emotional exhaustion, increased coping skills, and a positive residency experience in internal medicine residents [34, 35]. Post-operative coaching enhanced situational awareness, listening skills, and emergency decision-making in surgery residents [36]. This is the first study to our knowledge to examine the feasibility and acceptability of virtual coaching for residents on telemedicine encounters.

Regarding limitations and future directions, first, this pilot study analyzed a modest number of coaching sessions for residents from one specialty at a single institution. Second, only 42 % of the eligible residents completed fully virtual coaching sessions in the study period, which may be partially due to study onset and beginning of the COVID-19 pandemic corresponding to the launch of this coaching program, highlighting initial difficulties in scheduling observations within the limited study period and coaching to be lower priority at the time. Increasing resident buy-in by introducing potential benefits of the program early on, ensuring protected time for coaching, or mandating the communication coaching in the curriculum may be effective strategies to improve participation rate in the intervention. It is important to note that these early limitations have not persisted as the program has continued and grown.

Future directions include obtaining perspective of patients and clinic attendings, on the acceptability of the intervention, and demonstrating improved resident communication skills in this setting as rated by residents, faculty, or patients. However, with prior literature documenting the efficacy of coaching and the need for interventions toward resident communication in the new telemedicine sphere, demonstrating the feasibility and acceptability of this virtual coaching intervention is anticipated to be of interest to many residency programs.

Although self-assessments are broadly accepted tools in adult learning and medical education [37–39], they may be less accurate than expert evaluations, especially in those with less experience, lower training grade, and age [37–39]. Known as the Dunning-Kruger effect, individuals who lack knowledge and practice at a task tend to overestimate their performance and competencies and poorly identify areas for improvement [40, 41]. Trainees have been demonstrated to be better assessors in studies in which a grade was connected to the process [39], which was not a part of our intervention. Research also shows that nontechnical skills (such as communication skills) are more difficult to accurately self-assess than technical skills due to their complexity [37].

Given the modest number of coaching sessions, relatively small sample, and lower-than-expected participation rate, some potential biases may have occurred [42, 43]. Residents who completed a virtually coaching session might have been optimistic about coaching in general and thus more positively evaluated the intervention than those who did not participate. Subjects who were less likely to participate in the intervention for various reasons may be underrepresented in our study (selection bias). Since the intervention was conducted among the residents of one specialty only, the conclusions may not be relevant to all residents (interpretation bias). Therefore, we recognize that a communication coaching program may not be feasible for some residency programs. However, many elements of our intervention may be translatable. Residents, faculty, and program leadership may adapt coaching models in medical education [32, 34–36, 44], for example with residents consciously setting communication goals and actively reflecting on their performance after telemedicine encounters. Sharing these goals with clinic faculty supervisors and then inviting them to directly observe virtual encounters could facilitate targeted feedback related to the resident’s goals. Virtual coaching on communication skills in both the in-person and telemedicine settings may particularly benefit residents in challenging encounters such as those with cognitively impaired patients or with surrogate decision-makers.

## Conclusions

In conclusion, neurology residents and their faculty coaches found virtual coaching on communication skills within telemedicine encounters feasible and acceptable. Virtual coaching allowed our program to continue our educational mission in the face of major obstacles related to the pandemic. Elements of virtual coaching may be adopted to meet the proposed development milestones [5] and the goals of residents. Future studies of our coaching program will incorporate data on patient outcomes and resident communication performance, as these data will be critical for other residency programs considering implementation of a coaching program.

## Supplementary Information

**Additional file 1. **Resident Survey, Description of data: Survey distributed to residents.

**Additional file 2. **Coach Survey, Description of data: Survey distributed to coaches.

**Additional file 3. ** Feasibility acceptability, and perceived usefulness of virtually coaching residents on communication skills: findings differentiated between residents and coaches.

**Additional file 4. ** Faculty coaching training curriculum.

## Data Availability

The anonymized datasets used and analyzed during the current study are available from the corresponding author on reasonable request.
