# Supplementary material for: Feasibility and acceptability of virtually coaching residents on communication skills: a pilot study
Source: BMC Med Educ. 2021 Sep 29;21:513. doi: 10.1186/s12909-021-02936-w (PMC8478605; doi:10.1186/s12909-021-02936-w)
Supplement: Supplementary file 1 — Additional file 1. Resident Survey, Description of data: Survey distributed to residents. [file 12909_2021_2936_MOESM1_ESM.pdf]

Resident Survey

Evaluation of Communication Coaching on Telehealth Encounters Questionnaire

Resident Survey

Thank you for taking your time to participate in this survey. It will take approximately 5 minutes and will help us understand how our faculty coaches can best support resident coachees through telehealth coaching.

Please complete this survey as soon as possible after the debriefing session with your faculty coach / resident coachee. If you are a resident, you only need to complete it once. If you are a coach, you will be asked to complete the survey after each debriefing session on a telehealth encounter.

All responses are strictly confidential and de-identified. Results will only be reported in aggregate.

Thank you from the Coaching Initiative Steering Group.

Q1 Please indicate your role:

a. I am a resident
b. I am a faculty coach

Q2 Please indicate your department*

a. Neurology
b. Pediatrics
c. Surgery

Q3 What was the date of the telehealth encounter with a patient à pick the date

Q4 What was the setting of the telehealth encounter with a patient?

a. Resident Continuing Clinic (RCC)
b. Outpatient Elective
c. Clinic Block
d. Other, please describe _____________

Q5 What was the date of the debriefing session with your coach? à pick from calendar

The following questions are regarding your coach's direct observation of your telehealth encounter with a patient.

Q6 To what extend do you agree with the following statement for this session?

It was easy to schedule the coaching observation of a telehealth encounter.

not at all agree (1)	slightly agree (2)	moderately agree (3)	very much agree (4)	fully agree (5)

Q7 Did you experience any technical difficulties during the coaching observation of a telehealth encounter?

1. Yes
2. No à skip Q8

Q8 What technical difficulties did you experience during the coaching observation of a telehealth encounter? Please describe.

Q9 To what extent did the presence of a virtual coach disrupt the relationship you had with the patient during the telehealth encounter?

not at all disruptive	slightly disruptive	somewhat disruptive	very disruptive	extremely disruptive
(1)	(2)	(3)	(4)	(5)

The following questions are regarding the debriefing session with your faculty coach.

Q10 We debriefed using:

a. Phone (audio only)
b. Text à skip Q11
c. Facetime
d. Zoom
e. EPIC video
f. In person
g. Other, please describe ______________________

Q11 To what extend do you agree with the following statement for this session?

It was easy to schedule the debriefing session with my coach.

not at all agree (1)	slightly agree (2)	moderately agree (3)	very much agree (4)	fully agree (5)

Q12 To what extent do you agree with the following statements for this session?

1. The facilitated self-reflection with my coach was useful.
2. The feedback I received from faculty coach was useful.
3. My takeaways from this coaching session will be useful for my future telehealth encounters.

not at all agree (1)	slightly agree (2)	somewhat agree (3)	very agree (4)	extremely agree (5)

Q13 What are your feelings/reflections about being coached on telehealth encounters?

Q14 What are the challenges/drawbacks of being coached on telehealth encounters?

Q15 What are the opportunities/benefits of being coached on telehealth encounters?

*the survey was distributed to multiple departments
