# Supplementary material for: Feasibility and acceptability of virtually coaching residents on communication skills: a pilot study
Source: BMC Med Educ. 2021 Sep 29;21:513. doi: 10.1186/s12909-021-02936-w (PMC8478605; doi:10.1186/s12909-021-02936-w)
Supplement: Supplementary file 2 — Additional file 2. Coach Survey, Description of data: Survey distributed to coaches. [file 12909_2021_2936_MOESM2_ESM.pdf]

Coach Survey

Evaluation of Communication Coaching on Telehealth Encounters Questionnaire

Coach Survey

Thank you for taking your time to participate in this survey. It will take approximately 5 minutes and will help us understand how our faculty coaches can best support resident coachees through telehealth coaching.

Please complete this survey as soon as possible after the debriefing session with your faculty coach / resident coachee. If you are a resident, you only need to complete it once. If you are a coach, you will be asked to complete the survey after each debriefing session on a telehealth encounter.

All responses are strictly confidential and de-identified. Results will only be reported in aggregate.

Thank you from the Coaching Initiative Steering Group.

Q1 Please indicate your role:

a. I am a resident coachee
b. I am a faculty coach

Q2 Please indicate your department*:

a. Neurology
b. Pediatrics
c. Surgery

Q3 What was the date of the telehealth encounter with a patient à pick from calendar

Q4 What was the setting of the telehealth encounter with a patient?

a. Resident Continuing Clinic (RCC)
b. Outpatient Elective
c. Clinic Block
d. Other, please describe _____________

Q5 What was the date of the debriefing session à pick from calendar

The following questions are regarding the direct observation of the resident's telehealth encounter with a patient.

Q6 To what extend do you agree with the following statement for this session?

1. It was easy to schedule the coaching observation of a telehealth encounter.

not at all agree (1)	slightly agree (2)	moderately agree (3)	very much agree (4)	fully agree (5)

Q7 Did you experience any technical difficulties during the coaching observation of a telehealth encounter?

1. Yes
2. No à skip Q8

Q8 What technical difficulties did you experience during the coaching observation of a telehealth encounter?

Q9 To what extent did your presence disrupt the relationship the resident had with the patient during the telehealth encounter?

not at all disruptive	slightly disruptive	somewhat disruptive	very disruptive	extremely disruptive
(1)	(2)	(3)	(4)	(5)

The following questions are regarding the debriefing session with the resident coachee.

Q10 We debriefed using:

a. Phone (audio only)
b. Text à ship Q11
c. Facetime
d. Zoom
e. EPIC video
f. In person
g. Other: open text

Q11 To what extend do you agree with the following statement for this session?

It was easy to schedule the debriefing session with the resident.

not at all agree (1)	slightly agree (2)	moderately agree (3)	very much agree (4)	fully agree (5)

Q12 To what extent do you agree with the following statements for this session?

1. The facilitated self-reflection was useful for the resident.
2. The feedback I provided was useful for the resident.
3. The takeaways from this coaching session will be useful for the resident's future telehealth encounters.

not at all agree (1)	slightly agree (2)	moderately agree (3)	very much agree (4)	fully agree (5)

Q13 What are your feelings/reflections about coaching on telehealth encounters?

Q14 What are the challenges/drawbacks of coaching on telehealth encounters?

Q15 What are the opportunities / benefits coaching on telehealth encounters?

*the survey was distributed to multiple departments
