# Supplementary material for: Feasibility and acceptability of virtually coaching residents on communication skills: a pilot study
Source: BMC Med Educ. 2021 Sep 29;21:513. doi: 10.1186/s12909-021-02936-w (PMC8478605; doi:10.1186/s12909-021-02936-w)
Supplement: Supplementary file 3 — Additional file 3. Feasibility acceptability, and perceived usefulness of virtually coaching residents on communication skills: findings differentiated between residents and coaches. [file 12909_2021_2936_MOESM3_ESM.pptx]

## Slide 1
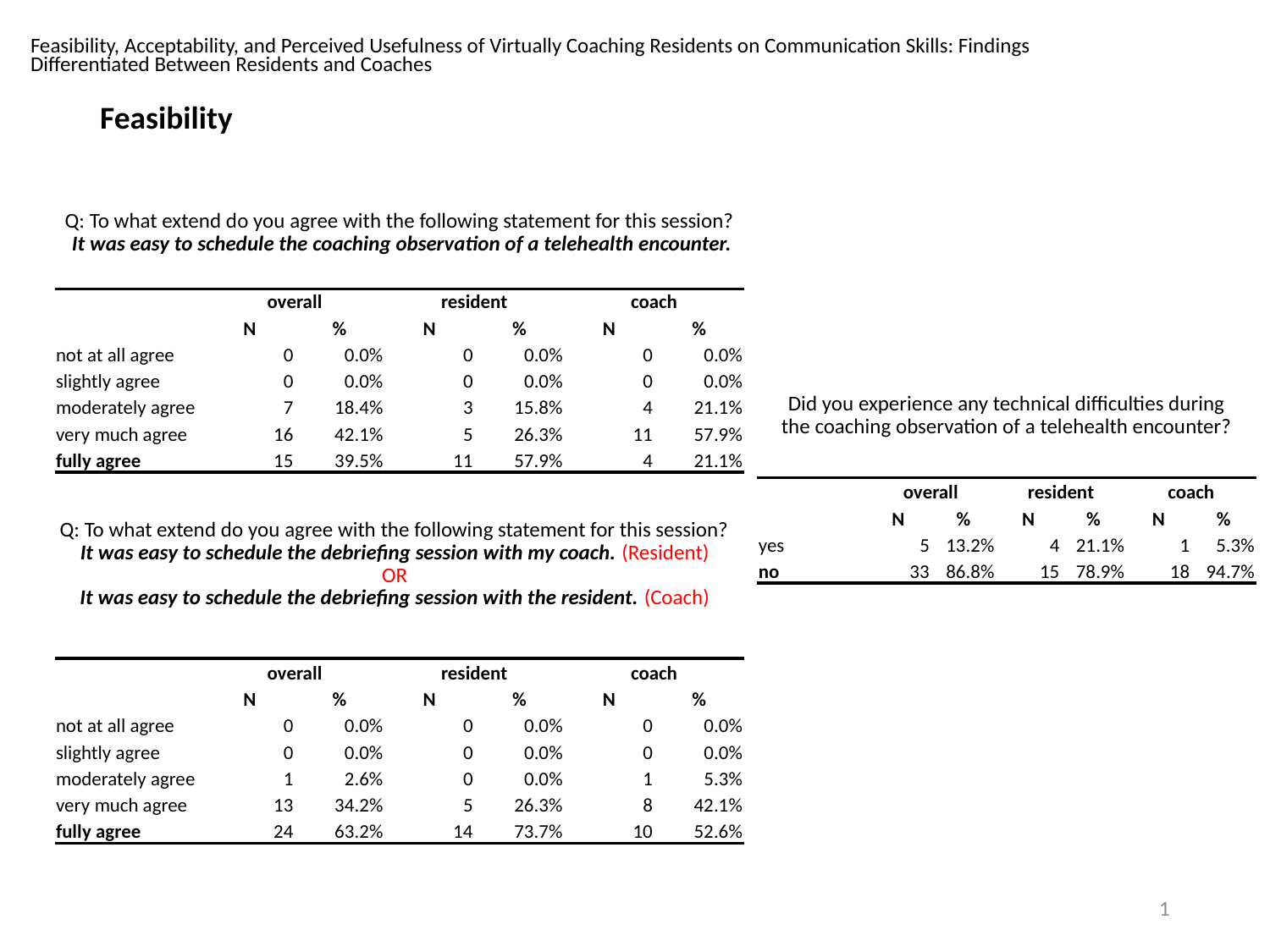

Feasibility, Acceptability, and Perceived Usefulness of Virtually Coaching Residents on Communication Skills: Findings Differentiated Between Residents and Coaches
# Feasibility
Q: To what extend do you agree with the following statement for this session?
It was easy to schedule the coaching observation of a telehealth encounter.
| | overall | | resident | | coach | |
| --- | --- | --- | --- | --- | --- | --- |
| | N | % | N | % | N | % |
| not at all agree | 0 | 0.0% | 0 | 0.0% | 0 | 0.0% |
| slightly agree | 0 | 0.0% | 0 | 0.0% | 0 | 0.0% |
| moderately agree | 7 | 18.4% | 3 | 15.8% | 4 | 21.1% |
| very much agree | 16 | 42.1% | 5 | 26.3% | 11 | 57.9% |
| fully agree | 15 | 39.5% | 11 | 57.9% | 4 | 21.1% |
Did you experience any technical difficulties during the coaching observation of a telehealth encounter?
| | overall | | resident | | coach | |
| --- | --- | --- | --- | --- | --- | --- |
| | N | % | N | % | N | % |
| yes | 5 | 13.2% | 4 | 21.1% | 1 | 5.3% |
| no | 33 | 86.8% | 15 | 78.9% | 18 | 94.7% |
Q: To what extend do you agree with the following statement for this session?
It was easy to schedule the debriefing session with my coach. (Resident)
OR
It was easy to schedule the debriefing session with the resident. (Coach)
| | overall | | resident | | coach | |
| --- | --- | --- | --- | --- | --- | --- |
| | N | % | N | % | N | % |
| not at all agree | 0 | 0.0% | 0 | 0.0% | 0 | 0.0% |
| slightly agree | 0 | 0.0% | 0 | 0.0% | 0 | 0.0% |
| moderately agree | 1 | 2.6% | 0 | 0.0% | 1 | 5.3% |
| very much agree | 13 | 34.2% | 5 | 26.3% | 8 | 42.1% |
| fully agree | 24 | 63.2% | 14 | 73.7% | 10 | 52.6% |
1

## Slide 2
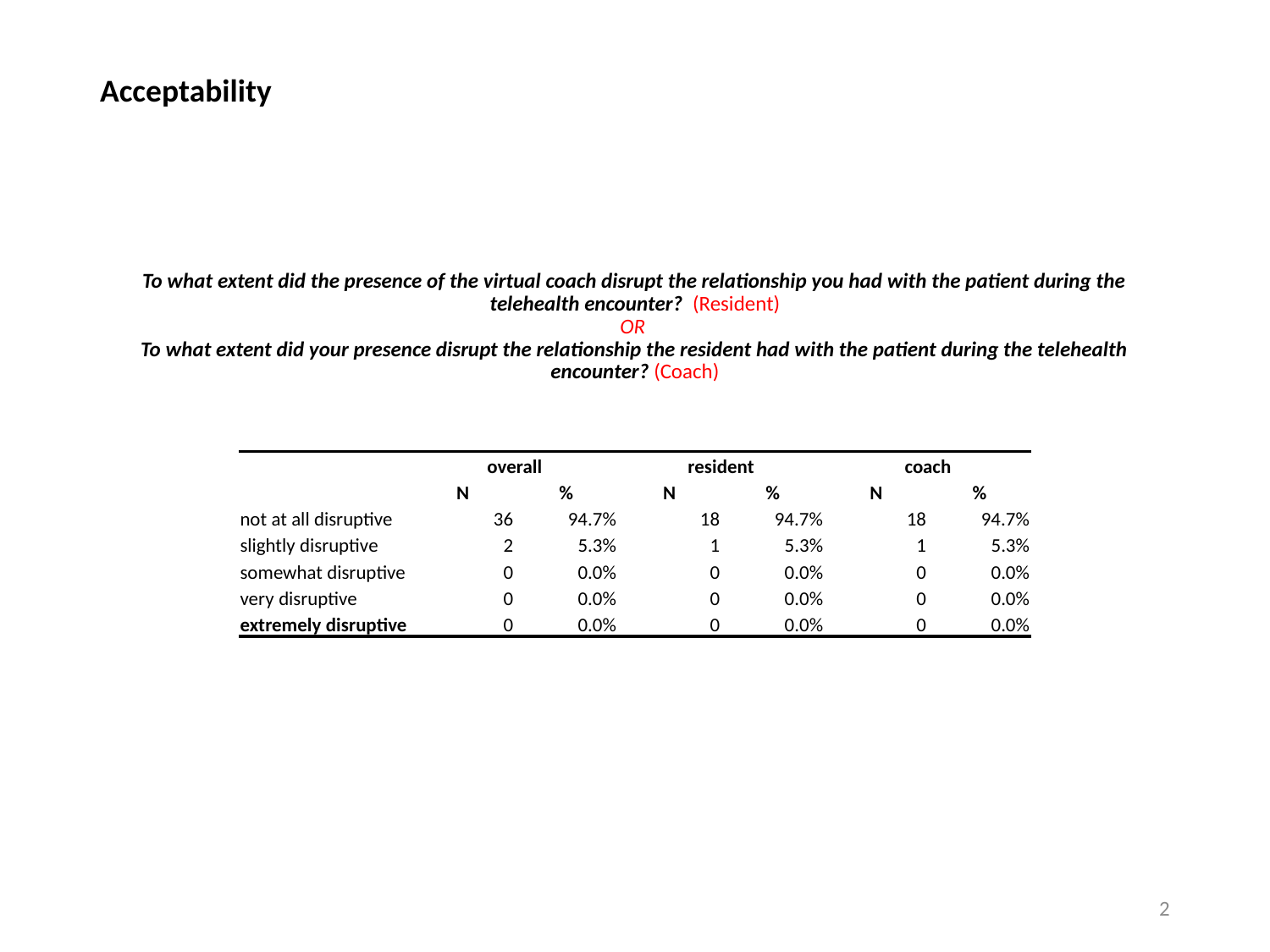

# Acceptability
To what extent did the presence of the virtual coach disrupt the relationship you had with the patient during the telehealth encounter? (Resident)
OR
To what extent did your presence disrupt the relationship the resident had with the patient during the telehealth encounter? (Coach)
| | overall | | resident | | coach | |
| --- | --- | --- | --- | --- | --- | --- |
| | N | % | N | % | N | % |
| not at all disruptive | 36 | 94.7% | 18 | 94.7% | 18 | 94.7% |
| slightly disruptive | 2 | 5.3% | 1 | 5.3% | 1 | 5.3% |
| somewhat disruptive | 0 | 0.0% | 0 | 0.0% | 0 | 0.0% |
| very disruptive | 0 | 0.0% | 0 | 0.0% | 0 | 0.0% |
| extremely disruptive | 0 | 0.0% | 0 | 0.0% | 0 | 0.0% |
2

## Slide 3
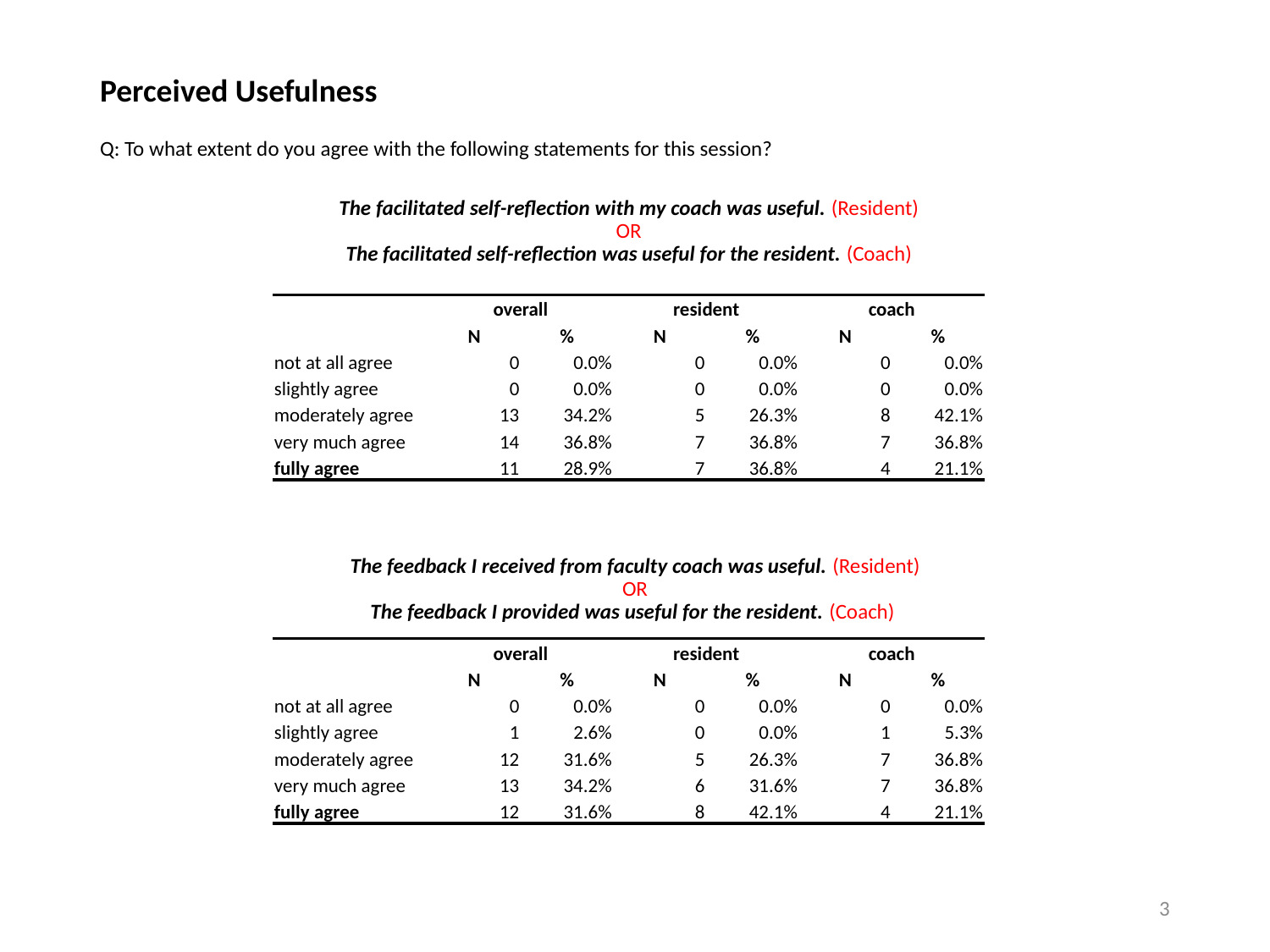

# Perceived Usefulness
Q: To what extent do you agree with the following statements for this session?
The facilitated self-reflection with my coach was useful. (Resident)
OR
The facilitated self-reflection was useful for the resident. (Coach)
| | overall | | resident | | coach | |
| --- | --- | --- | --- | --- | --- | --- |
| | N | % | N | % | N | % |
| not at all agree | 0 | 0.0% | 0 | 0.0% | 0 | 0.0% |
| slightly agree | 0 | 0.0% | 0 | 0.0% | 0 | 0.0% |
| moderately agree | 13 | 34.2% | 5 | 26.3% | 8 | 42.1% |
| very much agree | 14 | 36.8% | 7 | 36.8% | 7 | 36.8% |
| fully agree | 11 | 28.9% | 7 | 36.8% | 4 | 21.1% |
The feedback I received from faculty coach was useful. (Resident)
OR
The feedback I provided was useful for the resident. (Coach)
| | overall | | resident | | coach | |
| --- | --- | --- | --- | --- | --- | --- |
| | N | % | N | % | N | % |
| not at all agree | 0 | 0.0% | 0 | 0.0% | 0 | 0.0% |
| slightly agree | 1 | 2.6% | 0 | 0.0% | 1 | 5.3% |
| moderately agree | 12 | 31.6% | 5 | 26.3% | 7 | 36.8% |
| very much agree | 13 | 34.2% | 6 | 31.6% | 7 | 36.8% |
| fully agree | 12 | 31.6% | 8 | 42.1% | 4 | 21.1% |
3

## Slide 4
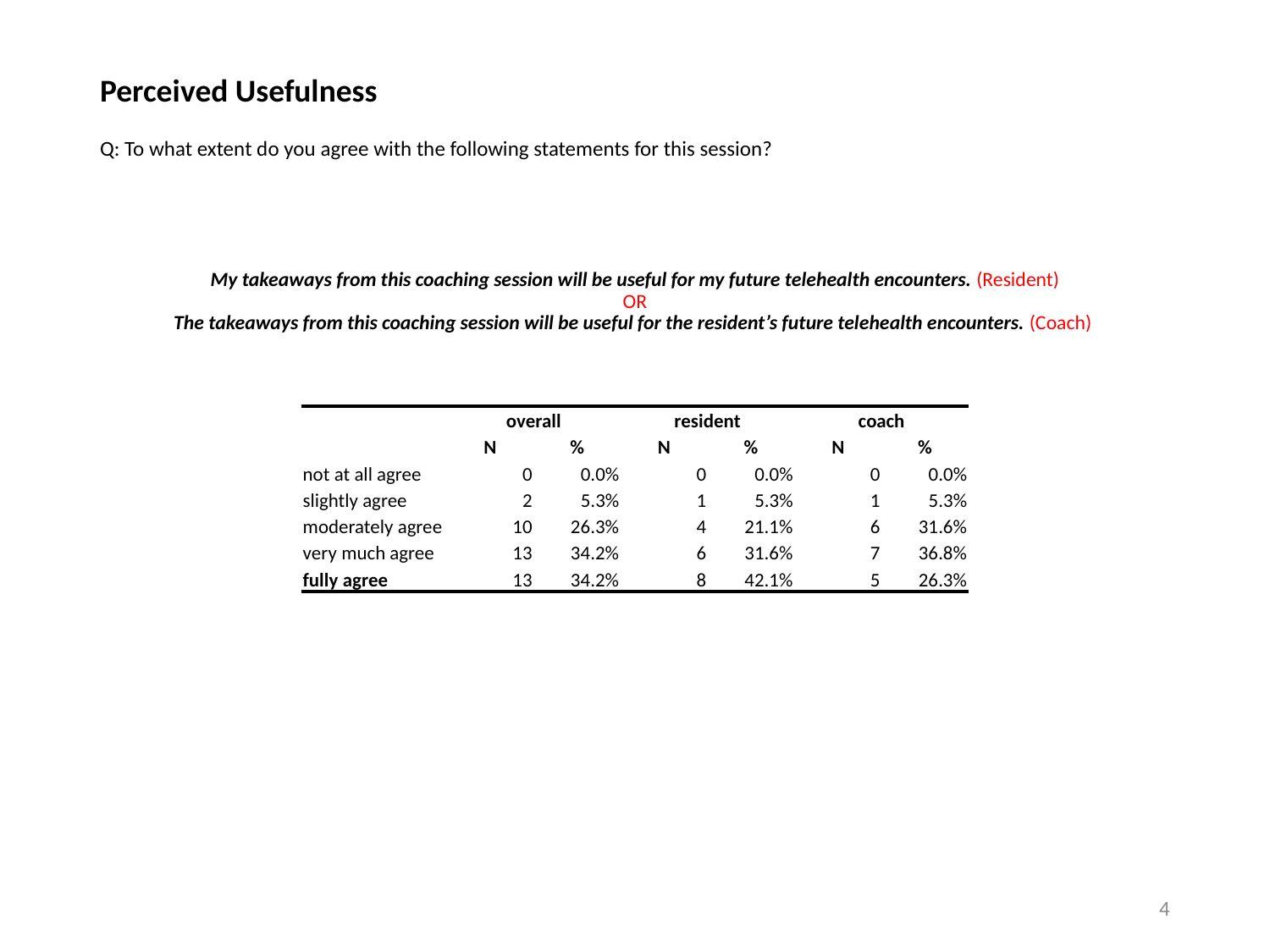

# Perceived Usefulness
Q: To what extent do you agree with the following statements for this session?
My takeaways from this coaching session will be useful for my future telehealth encounters. (Resident)
OR
The takeaways from this coaching session will be useful for the resident’s future telehealth encounters. (Coach)
| | overall | | resident | | coach | |
| --- | --- | --- | --- | --- | --- | --- |
| | N | % | N | % | N | % |
| not at all agree | 0 | 0.0% | 0 | 0.0% | 0 | 0.0% |
| slightly agree | 2 | 5.3% | 1 | 5.3% | 1 | 5.3% |
| moderately agree | 10 | 26.3% | 4 | 21.1% | 6 | 31.6% |
| very much agree | 13 | 34.2% | 6 | 31.6% | 7 | 36.8% |
| fully agree | 13 | 34.2% | 8 | 42.1% | 5 | 26.3% |
4
