# Supplementary material for: Feasibility and acceptability of virtually coaching residents on communication skills: a pilot study
Source: BMC Med Educ. 2021 Sep 29;21:513. doi: 10.1186/s12909-021-02936-w (PMC8478605; doi:10.1186/s12909-021-02936-w)
Supplement: Supplementary file 4 — Additional file 4. Faculty coaching training curriculum. [file 12909_2021_2936_MOESM4_ESM.pdf]

Faculty Coaching Training Curriculum

Faculty coaches participate in a half-day orientation presented by the leaders of the Stanford Pediatrics Coaching Program. Every month the coaches participate in 90-minute faculty development workshops on topics related to communication skills, coaching, and medical education delivered by experts from Stanford and other top institutions. The workshops bring together coaches from Neurology, Surgery, and Pediatrics.

Half-Day Orientation Agenda – February 2020

Communication Coaching Workshop
Stanford General Surgery, Neurology, & Pediatrics
February 24, 2020

**8:00-8:15am**	Arrival & breakfast
**8:15-8:30am**	Introductions
**8:30-9:15am**	Intro to Coaching, *led by Drs. Becky Blankenburg & Carrie Rassbach*<br>a. What is coaching? The sports to executive coaching spectrum<br>b. What is coaching? The mentoring to coaching spectrum<br>c. Coaching literature<br>d. Precision education: residency coaching programs
**9:15-9:45am**	General Principles in Feedback and Coaching, *led by Drs. Becky Blankenburg & Carrie Rassbach*<br>a. Facilitating reflection<br>b. Giving feedback<br>c. Goal-setting by asking coaching questions<br>d. Role play
**9:45-10:00am**	Break
**10:00-11:00am**	Year One: February-June 2020, *led by Drs. Carl Gold, Rebecca Miller-Kuhlmann, & Aussama Nassar*<br>a. How we got here<br>b. Focus of coaching sessions<br>c. Scheduling coaching sessions<br>d. Documenting coaching sessions in MedHub<br>e. Evaluation plan
**11:00-11:30am**	Demonstration of common & challenging coaching scenarios, *led by Pediatrics coaches*
**11:30-12:00pm**	Question and answer session with Pediatrics coaches, *moderated by Dr. Rebecca Miller-Kuhlmann*

2020-2021 Academic Year Faculty Development Workshops (in alphabetical order)

1. Active Listening
2. Bias
3. Coaching Integrating and Support within Residency Programs
4. Emotional Intelligence
5. Impact of Coach Role on Coaches' Learning and Engagement
6. Impact of Coach Role on Coaches' Wellbeing
7. Informed Consent
8. Managing Time Like a CEO
9. Routine Expertise vs Adaptive Expertise
10. Serious Illness Conversation Guide Training
11. Shared Decision Making
12. Stretching Great Learners
13. Teaching Generation Z
14. TeamSTEPPS
15. Telehealth Communication Skills
16. Telepresence 5
17. Wellness Toolkit
18. Wisconsin Surgical Coaching Program

More information about the coaching program and a list of future coaching development sessions may be found on this website:
https://med.stanford.edu/neurology/education/resident-coaching.html
